# The implications of exosomes in pregnancy: emerging as new diagnostic markers and therapeutics targets

**DOI:** 10.1186/s12964-022-00853-z

**Published:** 2022-04-12

**Authors:** Mehri Ghafourian, Roya Mahdavi, Zahra Akbari Jonoush, Mahvash Sadeghi, Nooshin Ghadiri, Maryam Farzaneh, Abdolah Mousavi Salehi

**Affiliations:** 1 Department of Immunology, School of Medicine, Ahvaz Jundishapur University of Medical Sciences, Ahvaz, Iran; 2 Fertility, Infertility and Perinatology Research Center, Ahvaz Jundishapur University of Medical Sciences, Ahvaz, Iran; 3 Student Research Committee, Ahvaz Jundishapur University of Medical Sciences, Ahvaz, Iran; 4 Cellular and Molecular Research Center, Medical Basic Science Research Institute, Ahvaz Jundishapur University of Medical Sciences, Ahvaz, Iran

**Keywords:** Extracellular vesicles, Exosomes, Pregnancy, Preeclampsia, Gestational diabetes mellitus, Down syndrome, Congenital heart disease, Neural tube defects

## Abstract

Extracellular vehicles (EVs) are a heterogeneous group of cell and membranous particles originating from different cell compartments. EVs participate in many essential physiological functions and mediate fetal-maternal communications. Exosomes are the smallest unit of EVs, which are delivered to the extracellular space. Exosomes can be released by the umbilical cord, placenta, amniotic fluid, and amniotic membranes and are involved in angiogenesis, endothelial cell migration, and embryo implantation. Also, various diseases such as gestational hypertension, gestational diabetes mellitus (GDM), preterm birth, and fetal growth restriction can be related to the content of placental exosomes during pregnancy. Due to exosomes' ability to transport signaling molecules and their effect on sperm function, they can also play a role in male and female infertility. In the new insight, exosomal miRNA can diagnose and treat infertilities disorders. In this review, we focused on the functions of exosomes during pregnancy.

**Video abstract**

**Supplementary Information:**

The online version contains supplementary material available at 10.1186/s12964-022-00853-z.

## Background

Extracellular vesicles (EVs) are small semipermeable membrane vesicles participating in intercellular communication. EVs participate in numerous biological processes. Inside the vesicles, a cargo is contained and guarded. The cargo presents the originating cell and pathophysiological states, e.g., in autoimmune sicknesses and cancer [1]. All cells, eukaryotes, prokaryotes, and eukaryotes release EVs as a part of their normal physiology during acquired abnormalities [2]. EVs can be classified by their biogenetic pathway, physical characteristics, and composition. There are three fundamental subgroups of EV, including apoptotic bodies, microvesicles, and exosomes. This division predates on the biogenetic mechanism of formation, release, and size [3]. Exosomes are a subset of EVs with a mean diameter of 30–120 nm [4]. Exosomes are released through an endosome-dependent pathway and deliver nucleic acids, proteins, lipids, cytokines, and metabolites [5]. They are releases by merging multicellular bodies containing intraductal vesicles with plasma membranes. They are also related to immune responses, viral pathogenesis, pregnancy, nervosum disease, and cancer progression [6, 7]. Several studies have shown that exosomes are relative to normal pregnancy and sophisticated pregnancy. During human pregnancy, maternal and fetal physiology is influenced by several factors [8]. The developing fetus and the placenta share their antigens to the mother [9]. Cellular communications between the mother and fetus occur by several secreted factors such as EVs, cytokines, and hormones [1]. Cellular communication is mainly mediated through direct cell-to-cell contacts, soluble factors, intercellular nanotubes, and EVs [10]. The placental assignments also include controlling and regulating the communication between the mother and the developing child. Placental cells of both maternal and embryonic origin, secrete not simplest soluble endocrine mediators however additionally extracellular vesicles, along with exosomes [1]. Exosomes play a critical role in pregnancy by modulating several processes including maternal immunologic response and metabolic adaptations. It identifies that the entire circulating exosomes in pregnancy increase across gestation [11–13]. Placenta, the principal organ in pregnancy, releases various hormones, growth factors, cytokines, miRNAs, and proteins which are crucial for maternal and fetal wellbeing placenta secretes large quantities of exosomes into the maternal circulation during normal and pregnancy complications like preeclampsia, gestational diabetes, fetal growth restriction, and preterm birth, and play important roles in several aspects of pregnancy including feto-maternalernal signaling [11]. Exosomes from other sources like follicular fluid, endometrium, embryo, and trophoblast cells may affect the feminine fertility, implantation, and early stages of pregnancy, whiles the placenta-derived exosomes are the most players in advanced stages of pregnancy. Contrary to growing interest in elucidating the role of exosomes during normal and sophisticated pregnancies, development within the field seems to be quite slow. On the opposite side, recent studies have demonstrated that pEXO (placenta-derived exosomes) has a key role within the establishment of maternal immune tolerance, which is critical for a successful pregnancy. Hence, to achieve a much better understanding of the underlying mechanism, we highlighted the advanced studies of pEXO on immune cells in pregnancy and the way this might result in complications of pregnancies with abnormal foetal developmental disorders [5, 14–16].

## Structure and physiological functions of exosomes

EVs are a heterogeneous group of cell and membranous particles originating from different cell compartments released outside the cell and retrieved in the body fluids and cell culture media [17]. EVs carry a variety of cargo, including RNAs, proteins, lipids, bioactive enzymes, molecules, molecular information, and DNA, and are released from all types of cells, including prokaryotic and eukaryotic cells [18]. EV secretion is a process that appears to be conserved throughout evolution [19]. According to their biogenesis, size, and release process, EVs can be classified into three main groups including, exosomes (originating from the endocytic pathway), microvesicles (MVs) (derived from plasma membrane), and apoptotic bodies (released by cell undergoing apoptosis) [20]. These classes contain different average size exosomes such as 30–120 nm [21], MVs (50–1000 nm) [22], and apoptotic bodies (50–5000 nm) [23] with a larger average size. Mammalian cells, including tumor cells, secrete EVs within a heterogeneous group of membrane vesicles, including both exosomes and MVs [22]. Exosomes were first identified in 1981, originally termed shedding vesicles, because of their 5′-nucleotidase activity, derived from various normal and neoplastic cell lines [24]. Recently, a new kind of non-membranous vesicles termed exomeres has been invested [25]. Exomeres are smaller than exosomes (> 50 nm) and cannot attach to the bilayer membrane [20].

The inner budding of endosomes generates exosomes, thereby can produce multivesicular bodies (MVBs) and intraluminal vesicles (ILVs) [26]. ILVs fuse with the plasma membrane and release exosomes into the extracellular space [27]. Plasma membrane- and cytosolic molecules-like nucleic acids, lipids, and proteins are endocytosed and transferred into early endosomes [28]. The endosome then matures, and early endosomes differentiate into late endosomes/ MVBs. Then, late endosomes fuse with lysosomes, leading to their degradation or fuse with the plasma membrane, releasing the vesicles into the extracellular space as exosomes [29, 30]. The biogenesis of exosomes is controlled by activating cell-specific receptors and signaling pathways [31]. The fusion of primary endocytic vesicles is the first step in the early endosome formation mediated by clathrin- or caveolin-dependent or independent pathways [32]. The Rab family of small GTPases controls different steps of vesicular trafficking. Rab27a and Rab27b were reported in MVE docking at the plasma membrane [33]. Rab27a has an important role in the size determination of MVEs, whereas Rab27b mediates the transfer of MVEs from microtubules to the actin-rich cortex and their maintenance at the cell periphery [34]. Ultimately, exosomes interact with recipient cells by direct signaling through ligand/receptor molecules on their respective surfaces or are taken up by recipient cells in unique fashion, such as direct membrane fusion, endocytosis, macropinocytosis, or even phagocytosis [35, 36]. The exosome formation pathway was regulated by the endosomal sorting complex required for transport (ESCRT)-dependent or an ESCRT-independent pathway [37]. The ESCRT is responsible for the accumulation and sorting of molecules channeled into ILVs. The main and critical ESCRT complexes such as ESCRT-0, -I, -II, and-III are responsible for the final delivery of ubiquitinated proteins to the degradation machinery [38].

Exosomes are highly enriched in ESCRT machinery-associated proteins (TSG101, HRS, and ALIX), which are involved in MVB synthesis [27]. Exosomes also express CD markers such as CD9, CD63, and CD81, which are used to recognize exosomes [39, 40]. Various metabolic enzymes, such as ATPase, glyceraldehyde-phosphate dehydrogenase (GAPDH), enolase 1, pyruvate kinase type M2 (PKM2), and phosphoglycerate kinase 1 (PGK1), have been detected in exosomes [41]. Heat shock proteins (such as HSP70 and HSP90) and MHC molecules are also found in exosomes derived from most cell types and are involved in antigen presentation [42]. Exosomes are involved in a variety of cellular biological processes. They have different roles and functions depending on what cell they originated [43]. The most important functions of exosomes are as follows. Absorption of unnecessary proteins and role in cellular maturation, dual function in the immune system [44, 45], development and normal physiology of the central nervous system [46], tissue regeneration, antigen presentation and induction of immune system responses [47], Influence on inflammatory responses, coagulation [48] and pregnancy [49] are the main biological functions of exosomes.

## Exosomes in pregnancy

### Origin and performance of exosomes in pregnancy

Exosomes are formed by the cellular endocytic pathway consisting of three different stages: (i) plasma membrane penetration from the endocytic vesicles; (ii) stage, inward budding of endosomal membrane starts, which gives rise the multivesicular bodies (MVBís); (iii) in the last stage, MVBís fuse with the plasma membrane and releases the vesicular contents (exosome) [50, 51]. Different lipidic molecules are known for their interference in exosome formation and release, like phosphatidic acid and ceramides. The size of the exosomes is erotic their site of origin and lipid bilayer structure in the cell [52]. Exosomes' origin and functions in pregnancy Exosomes play a vital role in pregnancy by modulating several processes, including maternal response and metabolic adaptations [11, 12]. it has been identified that the whole circulating exosomes in pregnancy increase across gestation [13] and increases in complications of pregnancy like Gestational DM (GDM) and pre-eclampsia (PE) [53]. Until now, the precise origin, cargo, and functions of those exosomes in maternal circulation do not seem to be fully understood and warrants further investigation. However, the placenta's principal organ in pregnancy secretes exosomes into the maternal circulation, and placental exosomes are detected as early as 6 weeks of pregnancy. Although the origin of microvesicles and exosomes is well known, the experimental discrimination of these vesicles types is difficult, so the terms are sometimes subsumed as extracellular Vesicles. Remarkably, exosomes derived from the placenta form a vital link by which the placenta communicates with other maternal tissues in normal and sophisticated pregnancies [13, 54].

### EVs in normal pregnancy in physiological pregnancy

EVs and exosomes particularly have time and again been indicated to act as components of fetal-maternal communication during implantation and placentation [55, 56]. while modulating the maternal reaction, maintaining cellular metabolic homeostasis, promoting fetal vasculogenesis, maternal uterine vascular adaptation, and preparing the uterus for delivery [57–60]. Attached to the uterus wall, the placenta constitutes the interface between the mother and fetus within the gestational period, ensuring gas exchange, nutrient and waste transfer, immunoglobulin transport, and hormone secretion. Maternal–fetal communication is feasible either through simple or facilitated diffusion, transport, or using EVs [61]. Through their content, embryonic EVs engulfed by specific maternal cells, find yourself regulating maternal adjustments.

Further on, placental EVs aid the pregnancy's vascular changes while also reflecting the placental function and fetal growth [62]. Placental-derived EVs distinguish themselves especially via their positivity for the syncytiotrophoblast (STB) marker placental alkaline phosphatase (PLAP), amongst other STB-derived EVs (STBEVs) [63], while both early and term placental cytotrophoblast cells are demonstrated to secrete, using exosomes, members of the B7 family of immunomodulatory molecules, namely B7-H1 (CD274), B7-H3 (CD276), and human leukocyte antigen-G5 molecules (HLA-G5) [64] Maternal and fetal exosome transfer in both directions has been demonstrated using fluorescently labeled exosomes in pregnant mouse models, thus reinforcing the isolation of exosomes from maternal blood samples as a non-invasive liquid biopsy [53]. Like other EVs, placenta-derived EVs are abundant in miRNAs, regulators of an organic phenomenon at the post-transcriptional level, exerting their effects by targeting multiple mRNAs [65]. By these means, miRNAs carried by EVs and transported to specific cells find yourself modifying the organic phenomenon pattern of the recipient cells. Among placenta-associated miRNAs, 46 miRNAs belonging to the chromosome 19miRNA cluster (C19MC) are identified expressed in villous trophoblasts [66]. Among these, miR-517b favors TNFα expression [67] while miR-516b-5p, miR-517-5p, and miR-518a-3p are shown to impact the PI3K-Akt and therefore the insulin signaling pathways, their expression levels being regulated by various stimuli, including oxidative stress and glucose levels [68]. Furthermore, in healthy oxen pregnancy models, placental exosome-derived miR 499 has been shown to downregulate NF-κB activation by targeting the Lin28B/let-7-ras signaling axis, therefore maintaining a small proinflammatory profile [69].

### EVs in spiral artery remodeling

Following successful implantation, decidualization, and placentation occurs, the resulting placenta ensures the mandatory resources for the optimal development of the embryo [70]. The nutrient supply is facilitated by the uterine spiral arteries, which undergo a considerable transformation under the influence of adaptive mechanisms distributed by cellular and molecular factors [71]. Specifically, both cellular and extracellular components of the maternal uterine spiral arteries undergo modifications like apoptosis, hyperplasia and hypertrophy, migration, and ECM remodeling, all under the rigorous coordination of invasive cytotrophoblast cells and decidual natural killer (NK) cells [72]. Trophoblast cells find themselves replacing the distal endothelial cells, acquiring a low-resistance vascular bed phenotype, fitting for unrestricted blood flow. During placental development, the migration of vascular smooth muscle cells (VSMC) plays a key role in spiral artery remodeling, a movement which has been demonstrated to be partly promoted by EVs released by extravillous trophoblast (EVT) cells via a unique EVT-VSMC exosomal communication pathway [73].

Furthermore, exosomal miRNAs and vascular endothelial protein A (VEGFA) are reported to be discharged by the implanted embryo to regulate blood flow [74]. based upon oxygen degrees, placental EVs have also been mentioned to stimulate vascular-angiogenesis, particularly in hypoxic situations [75]. On an identical note, Jia and colleagues have researched the role of maternal and duct blood exosomes on angiogenesis. By analyzing healthy pregnant women, they found that maternal and umbilical exosomes promoted human vein endothelial cells (HUVEC) proliferation and migration, together with angiogenesis, with 258 miRNAs being upregulated in both forms of exosomes [59]. Other trophoblast cells derived-EVs that have also been reported to possess pro-angiogenic effects by enhancing the proliferation of maternal endothelial cells via particular angiogenesis-related miRNA regulation are identified in fetal membrane blood [76].

## The importance of exosomes in pregnancy: focus on angiogenesis

In pregnancy, EVs can stimulate or impede angiogenesis based on their content and molecular expression, dependent on the cell of origin and its pathophysiological state [77]. Hypoxic conditions modulate the release of EVs from cytotrophoblast and placental cells in the first trimester of the pregnancy [78]. Exosomes can be released by the umbilical cord, placenta, amniotic fluid, and amniotic membranes [79]. During pregnancy, exosomes in amniotic fluid and urine induce tissue regeneration and angiogenesis [80]. Exosomes can produce pro-angiogenic factors that support normal gestation development and the maintenance of endothelial homeostasis [81]. The concentration of exosomes in maternal plasma increased more than two-fold towards the end of pregnancy [82]. It has been shown that exosomes' ability to modulate endothelial cell migration decreased over time [82]. Exosomes could originate from maternal circulation and cord blood and promote endothelial cell proliferation, migration, and tube formation [83]. The activity of maternal serum-derived exosomes is higher than that of exosomes from cord blood [84]. This is probably due to the differential expressions of miRNAs involved in regulating cell migration, such as miRNA-550a-5p, miRNA-122-5p, miRNA-210-3p, miR376c-3p, miRNA-151a-5p, and miRNA-296-5p [85]. Another source of the exosomes is human umbilical cord mesenchymal stem cells (UC-MSCs), which have pro-angiogenic activity [86]. UC-MSCs stimulates endothelial cell migration, proliferation, and angiogenesis in vitro [87]. Under hypoxic conditions, UC-MSC-exosomes have angiogenic potential in vitro. A large number of pro-angiogenic factors, including VEGF, VEGFR-2, MPC-1, angiogenin, tie-2/TEK, and IGF are present in these exosomes [88]. Moreover, upregulation of miRNA-150 was found in UC-MSC-derived exosomes from a healthy pregnancy in piglets. The miRNA-150 was associated with higher expression of VEGF and Notch1. Therefore, exosome miRNA-150 is a major regulator of angiogenesis in utero [88]. Various exosomal miRNAs, including miR486-1-5p and miR486-2-5p, are participated in migration, placental development, and angiogenesis [89].

## The roles of exosomes in embryo implantation

It has been investigated that the endometrial epithelium release EVs involved in the transfer of signaling miRNAs and adhesion molecules to the blastocyst and the adjacent endometrium into the uterine cavity [90]. It has been found that CD63+ and HSP70+ exosomes are present in the uterine luminal fluid (ULF) of cyclic and pregnant sheep [91]. Exosomes are involved in the suitable interaction between the embryo and uterine endometrium required for successful embryo implantation during pregnancy [92]. Endometrial epithelium can release exosomes into the uterine cavity and transfer specific miRNAs to the blastocyst or endometrial epithelial cells to enhance embryo implantation [93]. Comparing the miRNAs of the exosomes and their producer cells demonstrated that 13 of the 227 miRNAs were specific for exosomes and promoted embryo implantation [94]. Human endometrial epithelial cells-derived exosomes taken up by trophoblasts improve their adhesive features through enhanced focal adhesion kinase signaling [95]. Exosomal miR-30d is secreted by the human endometrium and upregulates genes that participated in embryo implantation, including integrin alpha-7, cadherin-5, and integrin beta-3 [96]. Extravillous trophoblast (EVT) invasion is one of the key steps for successful placentation [97]. There is differential expression of placenta-associated miRNAs (miR-520c-3p) between chorionic villous trophoblasts (CVTs) and EVTs [98]. The down-expression of endogenous miR-520c-3p results in accelerate CD44/hyaluronic acid (HA)-mediated EVT invasion [98]. Exosomal miR-520c-3p probably participates in CVT-EVT cell communication and EVT invasion [99]. Indeed, miR-520c-3p targeted CD44 in EVT, and downregulation of endogenous miR-520c-3p accelerated EVT invasion [100].

## Clinical application of exosomes and miRNAs in complicated pregnancies

Several studies reported that placental exosomes are involved in the pathology of pregnancy [101]. There is a connection between the quantity and content of placental exosomes and placental dysfunction, including gestational hypertension, PE, gestational diabetes mellitus (GDM), preterm birth, and fetal growth restriction [10, 101]. It has been found that the secretion of vesicles during pregnancies is complicated by gestational diabetes and preeclampsia [102]. Different concentrations of exosomal miRNA could be associated with these pathological states. Besides, cell-free DNA (cfDNA) of exosomal origin is considered a hallmark of pregnancy complications [103]. Fetal cfDNA, produced by apoptosis of placental cells in the trophoblast, is mainly released during normal pregnancies. However, the process of apoptosis increased during complicated gestation due to elevated levels of oxidative stress and inflammatory response. Therefore, alternation in the release of exosomes, their concentration and composition, and bioactivity are associated with pregnancy complications [12].

### Gestational hypertension

Gestational hypertension or pregnancy-induced hypertension is one of the most common complications in a pregnant woman after 20 weeks of gestation without the presence of protein in the urine. This disease is affecting up to 10% of pregnant women worldwide. There are 3 types of hypertensions in pregnancy, about 1% of pregnancies are affected by chronic hypertension (CHT), 5–6% by gestational or pregnancy-induced hypertension (GHT), and 1–2% by PE. In GHT, hypertension develops in the latter part of pregnancy without any other clinical symptoms of PE, which resolve postpartum [104]. The underlying pathophysiology of the disease is still elusive; nevertheless, it is thought to be related to a mechanism of altered trophoblast invasion and spiral artery remodeling. Eventually, the result prevents maternal blood flow to the placenta and leads to high perfusion pressure to the trophoblast layer. This phenomenon induces a cascade of inflammatory events, disrupting the balance of angiogenic factors, inducing platelet aggregation and injured trophoblast, all of which result in endothelial dysfunction manifested clinically as the preeclampsia syndrome. Moreover, followed by the release of possibly harmful materials such as cell fragments and EVs into the maternal circulation [105].

More than one thousand miRNAs are identified, which are expressed by different layers of the human placenta [106]. Also, some of these miRNAs are primate-specific and only expressed by placental and stem cells. Although the function of placentally expressed miRNAs is not fully understood, they appear to be involved in the regulation of placental development and play critical roles in normal physiology [107]. Moreover, it has been reported that either placenta-specific or not placenta-specific miRNAs are associated with pregnancy-related hypertensive diseases. Trophoblast-derived miRNAs are released to maternal circulation through the exosomal pathway [108]. Several investigations identified that the concentration of total peripheral blood exosomes was significantly increased in pregnant women with hypertensive disorders, which correlated with the severity of the diseases compared to normal pregnancies. Also, it has been exhibited that the contents of peripheral blood exosomes, especially miRNAs, are different between normal pregnancies and preeclamptic pregnancies and in different types of hypertension. In one study, plasma samples from women with CHT, GHT, PE, and normotensive pregnancies were collected. Exosomal miRNAs were extracted, and miRNA concentration was measured. The circulating exosomal total-miRNA and hsa-miR-210 were increased in women with PE [109]. Exosome-derived hsa-miR-210 may have a role in the pathomechanism of the disease [110].

Maternal plasma exosomal profiling of selected C19MC (chromosome 19 miRNA cluster) microRNAs also revealed the down-regulation of miR-517-5p, miR-520a-5p, and miR-525-5p was observed in patients with the later occurrence of GH and PE. It has been suggested that both exosomal miRNAs and proteins could employ as predictive biomarkers [111]. Moreover, another study found that the up-regulation of miR-516-5p, miR-517, miR-520 h, and miR-518b is associated with the later development of gestational hypertension. First-trimester screening of extracellular miR-520 h alone or combined with miR-518b identified a significant proportion of women with subsequent gestational hypertension [112]. Another study by Hromadnikova confirmed that the upregulation of miR-516-5p, miR-517, miR-520a, miR-525, and miR-526a is a characteristic of established preeclampsia and gestational hypertension [113]. Vascular dysfunction is believed to be primarily associated with hypertension. Ying et al. collected umbilical cord plasma samples from women with normal pregnancies and matched preeclamptic patients. Subsequently, they isolated circulating exosomes. They showed that umbilical cord plasma-derived exosomes from preeclamptic women drive vascular dysfunction by targeting 3-hydroxy-3-methylglutaryl-CoA synthase 1 (HMGCS1) in endothelial cells [114]. Therefore, the detection of peripheral blood exosomes and circulating miRNAs in gestation women is an important hallmark and is expected to identify early diagnostic markers for pregnancy-induced hypertension diseases [115].

### Gestational diabetes mellitus (GDM)

GDM is an alarming public health issue metabolic disorder, affecting approximately 9–15% of all pregnancies worldwide [92]. GDM is explained as any degree of glucose intolerance recognized for the first time during pregnancy. It is still unclear about the etiology of GDM, but some of the important factors in causing the GDM to include overweight status before pregnancy, ethnicity, previous GDM, hormone level, and family history of diabetes [116]. During pregnancy in the maternal peripheral blood, common factors that reflect pregnancy status are blood glucose glycocholic acid, soluble Fms-like tyrosine kinase-1, circulating foetal DNA and exosomes, and circulating miRNAs [117–119]. Exosomes can be extremely attention as they play a pivotal role in studying the pathophysiology of GDM and potential personalized therapy. Two separate studies performed by Saker et al. and Salomon et al. identified that the plasma concentration of exosomes is higher in normal pregnant women than in non-pregnant women, and placental exosomes are released into the maternal circulation at the beginning of 6 weeks of gestation. After delivery, the concentration of blood exosomes returns to nonpregnancy levels within 48 h [13, 82]. Different types of exosomes have been identified in pregnant according to their diverse origins. Most of these exosomes are derived from maternal cells, such as B cells, T cells, neutrophils, and endothelial cells [13, 120]. A small number of these exosomes originate from syncytiotrophoblasts, cytotrophoblasts, extravillous trophoblast cells, and placental vascular endothelial cells, with syncytiotrophoblasts being the main producers of placental exosomes [49, 121–123]. Nakahara et al. showed that placental-derived exosome (PdE) levels increased in all types of gestation, but GDM these levels were higher than in normal pregnancies. Also, increasing the level of PdE is significantly associated with maternal body mass index (BMI), glucose concentration, and fetal body weight, implying that exosomes can be involved in maternal metabolic adaptation to pregnancy and therefore that PdE may be used as an early predictor of adverse outcomes, including GDM and PE [124]. PdE in women with GDM may change maternal physiology by the process of exosomal placenta-maternal transfection, a “payload” of receptors proteins. Some mediators play a role in this system, including the vascular, pancreatic, adipose tissues, and innate immune [125]. GDM impacts trophoblasts or PD-MSCs, altering the endothelial activity supporting changes in transport glucose GLUT 3 and therefore delivery of energy substrates to the fetus [125]. PdE may play a role in proinflammatory response by producing adipokines associated with pregnancy and increased phenomenon under diabetic conditions. Jayabalan, N et al. showed that in GDM, exosomes secreted from adipose tissue may communicate with regulating placental glucose metabolism (glycolysis and gluconeogenesis) by improving the communication of adipose tissue-derived exosomes (exo-AT) to placental tissues, therefore, might become an active intervention strategy to prevent the consequences of GDM, such as fetal overgrowth [71, 126]. Rafał Sibiak et al., reported that placental exosomes isolated from the urine of patients with GDM carried significantly different amounts of several various microRNAs (miR-222-3p, miR-16-5p, miR-516 p, miR-517-3p, and miR-518-5p) compared with healthy controls and also) miR‒122-p; miR‒132-3p; miR‒1323; miR‒136-5p; miR‒182-3p; miR‒210-3p; miR‒29a-3p; miR‒29 bp; miR‒342-3p, and miR-520 h) was remarkably increased in GDM patients compared with normal controls [127]. the high levels of glucose and adipokines are considered factors that induce the overexpression of miR‑16‑5p. miR‑16‑5p is a modulator of the PI3K/Akt signaling pathway by regulating genes, such as Pi3Kr1 and Pi3kr3, mTOR, and Mapk3, as well as a result, of overexpression of these factors signaling pathways, has been connected with diabetes mellitus and GDM. Also, miR‑16‑5p control genes encode proteins 1 and 2 of the insulin receptor substrate (IRS1/IRS2). Therefore, the upregulation of miR‑16‑5p in patients with GDM will result in negative regulation of IRS1 and IRS2, which could lead to abnormal Wnt/β‑catenin signaling and, finally, diabetes [127–129]. The exosomal miRNAs hsa-miR-1910-5p, hsa-miR-16-5p, hsa-miR-92a-3p, and hsa-miR-92-3p can be upregulated expressed proteins in GDM, and their target protein respectively are 60S ribosomal protein L29 (RL29), serine/threonine-protein phosphatase 6 (PPP6), chloride intracellular channel protein 4 (CLIC4) and actin-related protein complex 2 (ARPC2). sEVs miRNAs regulatory STAT3 pathways associated with glucose metabolism and insulin signaling by targeted skeletal muscle. As a result of the Stat3 pathway, involved in the cytokine and nutrient-mediated insulin resistance in skeletal muscles [130]. Interestingly, sEVs miRNA- caused downregulation of STAT3 and decreased insulin signaling in skeletal muscles has been reported in type 2 diabetes [130]. furthermore, STAT3 acts as a link between obesity and diabetes by mediating lipid-induced insulin resistance. In this sense, the differential miRNA profile in exosomes and their target proteins in the skeletal muscles may contribute to the pathophysiology of GDM [131, 132]. Another example of the exosomal miRNAs that can be affected in skeletal muscle cells is miR-92a-3p which induces SOCS2 and suppress NOS2 expression. SOCS proteins can downregulate the cytokine or tyrosine kinase receptor signaling by targeting proteins such as JAK and IRS family members [133]. Increased proinflammatory cytokines such as IL-1β, IL-6, TNF-α, and growth hormone play a role in peripheral insulin resistance [134]. It has been reported that SOCS-1 and SOCS3 are associated with insulin resistance by negative regulation of IRS-1 and IRS-2.Another study demonstrated that the increase (miRNA-518d) might contribute to the pathology of the development of GDM by the effect on the regulation of proliferator-activated receptor (PPAR) expression [135]. miR-222 from adipose tissue can be regulated ER-expression in estrogen-induced insulin resistance in GDM. Exosomal miRs can be profiled in biomarker discovery studies. Therefore, via the study of these exosomal miRs, important aspects could be better understood and explored for more effective future diagnoses and new therapeutic approaches to GDM [136].

### Preeclampsia (PE)

PE is a condition associated with common complications of pregnancy characterized by high blood pressure and lead to damage to another organ system, especially the liver and kidneys. Preeclampsia usually appears during the second half of pregnancy in women whose blood pressure has been normal. The main theoretical underlying the etiology of PE centers on abnormal placentation due to insufficient spiral-artery remodeling caused by poor trophoblast invasion. Increased placental oxidative stress is elicited by a predisposing condition, which stimulates the release of microvesicles from the syncytial layer of the placenta. PE is defined as a blood pressure reading of 140/90 mm Hg or more after 20 weeks of gestation in a woman who previously had normal blood pressure and has proteinuria (0.3 g protein in 24-h urine collection). Preeclampsia symptoms include: facial or hand swelling, Severe headaches that persist, vision alters, Breathing difficulties, Gaining a significant amount of weight in a short period, Pain in the abdomen or shoulders. During the second half of pregnancy, women may experience nausea or vomiting [137–141]. The disease causes significant fetal and maternal mortality. The number of women and babies who die of these disorders, approximately 76,000 dies of PE, is thought to be 500,000 per annum, respectively. The newborn can lead to a higher risk of short-term and long-term disease in infancy, childhood, and adulthood. Currently, there is no helpful treatment to prevent the long-term consequences of PE, mainly because the pathophysiology is not well understood and is not detected early enough [142–145]. maternal factors, blood pressure, proteinuria, and uterine-artery Doppler velocimetry are an example of clinical markers that remain the most reliable methods in PE monitoring and helpful care of the mother and fetus [146, 147]. Nevertheless, they cannot be used in the early diagnosis of PE. However, they may still aid as useful markers when combined biomarkers of PE include cytokines, proteins, angiogenic and antiangiogenic factors, which have a vital role in the pathogenesis and etiology of PE [148].

Exosomes secreted from the placenta to the peripheral circulation can be detected from the plasma of pregnant women after 6 weeks of pregnancy and may be involved in the pathogenesis of preeclampsia. Researchers pursued circulating exosomes in the many studies, isolated from women with preeclampsia and compared with exosomes from women with uncomplicated term pregnancies. Li, H., et al. reported that the concentration of plasma exosomes from women preeclampsia was 1.47-fold and 1.45-fold higher, respectively, compared with healthy controls. The profile of plasma exosome concentration across a range of particle sizes was determined using nanoparticles, and, Interestingly, the mean diameter of exosomes derived from participants with preeclampsia (107.5 nm) was larger than that of controls. They found miR-153-3p and miR-325-3p exhibited a twofold upregulation in exosomes from preeclampsia, but this observation's biological significance remains uncertain [149]. Zeng et al. and Liang et al. showed that overexpression of miR-153-3p inhibited cell proliferation and invasion and promoted apoptosis.

Furthermore, miR-153 can bind the 3′untranslated region of Hypoxia Inducible Factor 1 Subunit Alpha mRNA and inhibit its expression associated with diminished tube formation in primary human umbilical vein endothelial cells reduced Vascular Endothelial Growth Factor A expression, and angiogenesis [150–152]. Another exosome isolated from women preeclampsia, such as placental miR-342-3p, can be upregulated in preeclamptic and implicated in endothelial cell dysfunction in obese children [153, 154]. Salomon, C. et al. suggested that the concentration of total exosomes and placental exosomes present in maternal circulation increased during normal and PE pregnancies. Remarkably, the concentration of exosomes was higher in PE than normal pregnancies matched by gestational age. The other result from these studies suggests that, in early pregnancy (i.e., 11–14 weeks), presymptomatic women who develop PE can be recognized by the concentration of exosomes within their plasma [53]. exosomes from the first trimester of pregnancy are more potent in promoting endothelial cell migration. Also, they can be involved in the inflammatory response, vasculogenesis, and angiogenesis, so exosomes from preeclampsia are suggested to contribute to the dissemination of endothelial damage by sequestering the free vascular endothelial growth factor (VEGF) in the maternal circulation [155]. Besides, proteins such as syncytin-1 and syncytin-2 contributed to EVs fusion with a target cell in placental development, and human trophoblasts fusion is downregulated in placenta-derived exosomes and trophoblasts from preeclampsia [156, 157]. To date, little is known about the exosome profile during PE pregnancies. However, the motivation for presenting exosomes as a candidate biomarker of PE is yet to be estimated in terms of the FDA-approved biomarker criterion [158].

### Preterm birth (PTB)

Normal Parturitionis an inflammatory process involving both fetal and maternal tissues; therefore, the inflammatory signals of fetal lead to functional progesterone withdrawal, the increase of inflammatory factors such as cytokines and chemokines mediated by the proinflammatory transcription factor NF-κB (nuclear factor κB) in the uterine cavity and activation of immune cells which disrupts the homeostatic factors that maintain pregnancy and leads to the promotion of fetal delivery (i.e., around 37–40 weeks of gestation). There are common causes of labor signaling chiefly related to fetal and maternal endocrine changes, including cortisol production, functional progesterone secretion, and immune changes such as leukocyte infiltration into embryonic connective tissues associated with fetal development [159–164]. If, for any reason, the balance between endocrine functions and the immune system is disturbed leads to inflammatory overload that interferes with the maintenance of pregnancy and therefore changes related to childbirth [67]. A Clear understanding of these signals and their mechanisms in normal pregnancies can provide vision into the pathological activation of these signals, leading to spontaneous preterm delivery. Fetal endocrine signals are mostly considered to contribute to the timing of birth. Preterm birth is a major pregnancy complication that affects 15% to 20% of all pregnancies worldwide [165]. Preterm births happen before 37 weeks gestation, with very preterm infants born at 38 before 32 weeks of pregnancy. Preterm delivery leads to low birth weight, respiratory distress syndrome, gastrointestinal, immunologic, central nervous system, hearing, vision problems, disruption of immune homeostasis, and overwhelming inflammation due to either infection or other noninfectious risk factors before 37 weeks, often resulting in spontaneous PTB. The risk factors for preterm birth include maternal nutritional status, maternal obesity, ethnicity, socioeconomic status, smoking, maternal age, parity, multiple pregnancies, and Intrauterine infection [166]. We can use extracellular vesicles specifical exosomes to understand the paracrine signaling mechanism. Menon, R., et al., selected four groups, including term not in labor (TNIL), term in labor (TL), preterm premature rupture of membranes (pPROM), and PTB. They studied changes in maternal plasma exosome proteomics contents in term and preterm births. Exosomes Were evaluated in size, morphology, quantity, and markers. To determine the protein profile in exosomes, they used quantitative and information-independent methods [67]. Pathway genius analysis determines pathways related to protein profiles identified in exosomes. The MP exosomes were spherical, averaged 120 nm in diameter, and the exosomal proteins CD63 and TSG101 were positive regardless of pregnancy status. No significant changes in exosome values ​​in maternal circulation were observed between groups. SWATH-MS identified 72 statistically significant proteins among the study groups. Bioinformatics analysis showed that the proteins in the exosomes in the TNIL, TL, pPROM, and PTB target pathways are mainly associated with inflammatory and metabolic signals. Exosomal data suggest that homeostatic imbalances, particularly inflammatory signaling and endocrine disorders, may impair pregnancy maintenance and lead to term and preterm labor changes. Reflection of physiological changes in exosomes indicates its usefulness as biomarkers and indicators of cellular function [67]. Sheller-Miller, S. et al. reported that one of the effective factors in the pathophysiology of preterm labor (PTB) was the accumulation of immune cells and the activation of the proinflammatory transcription factor NF-κB in maternal–fetal uterine tissues. One way to decrease infection-related PTB was to reduce the fetal inflammatory response and activate NF-κB. Thus, they engineered extracellular vesicles (exosomes) that contained an NF-κB inhibitor, called the supernatant (SR) IκBα. Treatment mice model with SR exosomes (1 × 10^10^ per intraperitoneal injection) after lipopolysaccharide (LPS) challenge on day 15 of gestation (E15), prolonged gestation over 24 h (PTB ≤ E18.5), and reduction of maternal inflammation (n n 4) observed. Besides, using a transgenic model in which fetal tissues express the fluorescent red protein tdTomato while maternal tissues do not, they reported that LPS induced PTB in mice with cell invasion. The innate immunity of the fetus, not the mother, is related to the uterine tissues of the mother. SR packaged in exosomes provides a stable and specific intervention to reduce the inflammatory response associated with PTB [167]. Sheller-Miller, S. et al. worked in mice model. In this experiment, it was reported that endocrine factors and maturation signals of fetal organs determine the time of birth. They isolated maternal plasma exosomes from CD-1 mice identified during pregnancy, and the biological pathways associated with the loaded proteins were expressed differently. The results showed that the shape and size of the exosomes did not change during pregnancy. However, a gradual increase in exosomes carrying inflammatory mediators was shown from a day of gestation (E) 5 to E19. Also, the effects of plasma exosomes in late pregnancy (E18) from uterine fetal uterine tissues on delivery were determined. Intraperitoneal injection of E18 exosomes into E15 mice located in maternal reproductive tissues and embryonic parts of the uterus. Compared to the timely control group, preterm delivery occurred in exosome-treated mice at E18 and raised inflammatory mediators at E17 in the fetal cervix, uterus, and fetal membrane but not in the placenta. This effect was not observed in injected mice with early pregnancy exosomes (E9). This study shows evidence that exosomes act as mediators of paracrine delivery and delivery [68].

Menon, R. et al. studied changes in the concentration of exosomal miRNA in maternal plasma between parturient mothers and preterm infants during pregnancy using a longitudinal study design. In this descriptive study, miRNA content in exosomes present in maternal plasma at preterm and PTB (20 and n = 10 in each pregnancy, respectively) during pregnancy and delivery time. They were determined changes in exosomal miRNA in maternal plasma during a term and gestational pregnancy using Sequences of 75 cycles with high output Next Seq 500 a platform. A total of 167 and 153 miRNAs showed significant changes as a function of gestational age during pregnancy and PTB, respectively (P < 0.05). Remarkably, comparative analysis between exosomal miRNA profiles between term and PTB reported a total of 173 miRNAs that changed significantly (P < 0.05) during pregnancy. Specific trends in changes (e.g., increase, decrease, and both) were also identified as a function of gestational age. Using bioinformatics analysis, they showed that differences in miRNA profiles target the signaling pathways associated with TGF-β, p53, and glucocorticoid receptor signaling, respectively. They suggested that the miRNA content of exosomes in the maternal circulation may indicate the biomolecular "fingerprint" of pregnancy progression [168]. A literature review using extracellular vesicles and preterm birth, exosomes, and preterm birth yielded only a few articles from the past decade. This indicates that studies on EVs' role in preterm birth are still limited. Gray, C. et al. revealed that circulating miRNAs, such as miR-302b, miR-548, and miR-1253, are downregulated and that miR-223 is upregulated in plasma from those with spontaneous preterm birth compared to those with normal pregnancy [169].

### Fetal growth restriction

Most pregnant women have a natural pregnancy and delivery, but some of them experience abnormalities during pregnancy that affect their health or the fetus's health, such as fetal growth restriction that affects 7–10% of all pregnancies [170, 171]. Anomalies of the neural tube, congenital heart disease, and other anomalies affect the fetus. Fetal growth restriction (FGR) is a disease in which a genetically specified fetal potential is stunted during pregnancy [172]. Abnormal fetal growth can be caused by maternal smoking, starvation, illness, or congenital disabilities [173]. FGR is most commonly associated with preeclampsia and accounts for roughly one-third of premature births. FGR is diagnosed by high-quality ultrasound programs that detect fetal weight less than the 10th percentile for gestational age [174]. The placenta is a crucial organ for fetal growth and development. FGR is a placental abruption induced by a trophoblast regulatory defect that produces uterine insufficiency [175]. Exosomes may play a function in pregnancy-related issues such as fetal growth limitation.

In a cohort study, Miranda, J. et al., 10 commonly grown fetuses, and 20 small fetuses were sub-classified into SGA and FGR, consequently to birth weight (BW) percentile and fetoplacental Doppler. Exosomes were isolated from maternal and fetal plasma and characterized by morphology, enrichment of exosomal proteins, and size distribution by electron microscopy, western blot, and nanoparticle tracking analysis, respectively. Total and specific placenta-derived exosomes were determined using quantum dots coupled with CD63 + ve and placental-type alkaline phosphatase (PLAP) + ve antibodies, respectively showed. The placental-type alkaline phosphatase (PLAP) + exosome ratio in FGR was lower than in the control group and SGA cases [171].

Another cohort study included women that later developed FGR (n = 63), and 102 controls by Hromadnikova, I., et al. they performed profiling Maternal plasma exosome with the selection of C19MC microRNAs with diagnostical potential only (miR-516b-5p, miR-517-5p, miR-518b, miR-520a-5p, miR-520h, and miR-525-5p) using real-time RT-PCR. MiR-520a-5p Represents a Novel Maternal Plasma Exosome C19MC MicroRNA Biomarker revealed a novel down-regulated during the first trimester of gestation for women destined to develop FGR [60].

Hromadnikova et al. showed that upregulation of miR-499a-5p is a common feature of all placental insufficiencies such as preeclampsia, gestational hypertension, and FGR; they also demonstrated an upregulation miR-1-3p in FGR pregnancies with abnormal umbilical fetal flows. they founded some of a series of miRNAs such as (miR-16-5p, miR-26a-5p, miR-100-5p, miR-103a-3p, miR-122-5p, miR-125b-5p, miR-126-3p, miR-143-3p, miR-145-5p, miR-195-5p, miR-199a-5p, miR-221-3p, miR-342-3p, and miR-574-3p) that downregulation and finally the delivery before 34 weeks of gestation [176]. Xiaotao et al. investigated microRNA-210 via the CRISPR/Cas9 technology to develop a miR-210-deficient mouse strain with dramatically reduced miR-210 expression in several tissues. After miR-210 was deleted from mice, little effect on the reproductive rate and litter size was detected. Continuous treatment of pregnant mice to hypoxia (10.5 percent O2) from E6.5 to E10.5 or E18.5 resulted in fetal weight loss, significantly aggravated in dams lacking miR-210. When hypoxia was applied to knockout mice, analysis of the placental structure revealed that the placental spongiotrophoblast layer expanded less, and the formation of labyrinth fetal blood arteries was impaired compared to wild-type controls. They discovered that miR-210 regulates placental adaptation to hypoxic stress during pregnancy and reduced miR-210 expression ultimately results in fetal growth restriction [177]. According to genome-wide expression profile research in placental tissue, several miRNAs implicated in collagen and growth factor signaling are connected with the infant's birth weight. Another study reported on a genome-wide examination of the IUGR placenta. 37 differentially expressed miRNAs, four of which belong to the C19MC (520a-3p, 520 f-5p, 515–5p, and 519–5p) and six of which belong to the C14MC (299–3p, 494–3p, 376a-5p, 382–3p, 154–3p, and 369–3p), are implicated in the regulation of cell migration and proliferation [178]. The researchers discovered that the expression of miR-518b is lowered in Intrauterine Growth Restriction( IUGR) instances, whereas the expression of miR-519a, another miRNA implicated in placental functions, is elevated in IUGR cases, according to their findings. This collection of studies suggests that aberrant expression of these microRNAs is connected with impaired placental trophoblast activity, which results in fetal growth restriction [179].

#### Down syndrome

Down syndrome (DS) is the most prevalent genetic, developmental, and multisystem impairment caused by the replication of chromosome 21 (Hsa21), impacting approximately one in every 800 live births worldwide [180]. These traits are associated with DS: mental impairments, congenital cardiac disease, hypotension, muscle weakness, and other developmental abnormalities [181]. This syndrome also involves imaging the cerebral cortex, hippocampus, and cerebellum. The volume of gray matter in the forehead of DS patients continues to diminish throughout puberty [182]. The studies indicate that primary cortical damage occurs prior to phenotypes such as AD in adult DS patients, demonstrating that regions of the brain are affected by DS. The APP gene is located on chromosome 21, and individuals with DS build dangerous amyloid peptides in their early years [183] and amyloid plaques in their third or fourth decade [184]. Recent evidence indicates that overexpression of the APP gene is required to develop AD in patients with Down syndrome [185]. While advancements in technology, medical therapy, and social initiatives have increased life expectancy, many of the underlying causes of this disorder remain unexplained. Exosomes are one method for investigating DS. They may identify novel neurodegenerative and disease biomarkers. Transsynaptic transmission of pathogenic components is also implicated in AD and other neurological diseases [186]. Exosomes are a helpful marker for elucidating the etiologies of DS. Exosomal screening may shed light on the persistence of proteins produced by neurons. Results can be obtained using the same exosomal markers from serum or plasma samples obtained from a consortium of biobanks. The exosome study enables reliable assessment of treatment efficacy [186]. In DS and AD patients, endosomal pathways are dysfunctional, with abnormally enlarged early endosomes in neurons. Gauthier et al. reported that endosomal MVBs could release endosomal material into the extracellular environment via exosomes. They found increased exosome secretion in the brains of DS patients, a mouse model, and DS fibroblasts. They also found increased exosome regulator tetraspanin CD63 in DS brains. Significantly, CD63 knockdown reduced exosome release and aggravated endosomal disease in DS fibroblasts. This study found that increasing exosome secretion reduces endosomal abnormalities in DS [187]. Erturk et al. evaluated the use of 14 miRNAs on chromosome 21 in prenatal DS testing. They chose 56 patients, 23 with Down syndrome fetuses and 33control cases. The research and control groups had pregnant women in their 17th and 18th weeks. Real-time RT-PCR was used to assess miRNA expression. MiR-3156 and miR-99a raised maternal plasma levels in women carrying Down syndrome fetuses [188]. CD81 levels were more significant in DS neuron-derived exosomes than in control populations by Hamlett et al. Regardless of ag, neuron-derived exosomes were more plentiful in DS blood samples [189].

Another study by Zbucka-Kretowska et al. indicated that 6 miRNAs were overexpressing (hsa-miR-15a, hsa-let-7d, hsa-miR-142, hsa-miR-23a, hsa-miR-199, hsa-miR-191) and 7 were downregulated (hsa-miR-1290, hsa-miR-1915, hsa-miR30e, hsa-miR-1260, hsa-miR-483, hsa-miR-548, hsa-miR-590) in plasma samples of women with fetal DS syndrome. The data results that identified miRNAs may attend to the pathogenesis of DS and would potentially play a significant role in future preventive therapies [190]. The action of miR-138-5p and the downregulation of its target, enhancer of zeste homolog 2, in the hippocampus may be involved in the intellectual handicap of DS patients, according to Shi et al. [191]. Karaca, Emin et al. tested 56 pregnant women; 23 had Down syndrome fetuses, and 33 had normal karyotypes. Invasive prenatal testing was indicated by maternal age and increased risk of Down syndrome in screening tests. The study and control groups were between 17- and 18-weeks’ gestation. A real-time polymerase chain reaction was used to evaluate microRNA expression. The study group had higher expression levels of microRNA-125b-2, microRNA-155, and microRNA-3156 than the control group [192]. Zbucka-Kretowska, M measured miRNA expression in plasma of pregnant women with Down syndrome (DS). They used NanoString technology to evaluate the expression level of 800 miRNAs in 198 amniocentesis conducted at 15–18 weeks gestation on 12 patients with foetal DS and 12 uncomplicated pregnancies who delivered healthy newborns at term. They found 6 miRNAs elevated (hsa-miR-15a, hsa-let-7d, hsa-miR-142, hsa-miR-23a, hsa-miR-199, hsa-miR-191) and 7 miRNAs downregulated (hsa-miR-1290, hsa-miR-1915, hsa-miR-30e, MiRNA-regulated genes are implicated in CNS development, congenital abnormalities, and heart problems. The results of this work revealed a DS-specific miRNA expression signature, which can be used to develop a non-invasive miRNA panel for DS diagnosis. Scientists believe that discovered miRNAs may play a role in DS development and could be used in future preventive therapeutics [193].

#### Congenital heart disease

More than half of all cardiovascular events in men and women under 75 years are caused by coronary heart disease (CHD) [194]. CHD is the most common human congenital disability and the leading cause of perinatal mortality [195]. Around 0.8–0.9% of newborns occur, and prevalence increases worldwide [196]. The mortality incidence rate from congenital heart disease was 81 cases per 100,000 live births. Most women with heart disease can have a successful pregnancy. Nevertheless, the highest mortality rates are seen in women with normal heart structures who did not have heart disease before pregnancy [197]. There are no specific symptoms of CHD, but complications such as Mental retardation, preterm delivery, and even fetal and neonatal death are more common among children of women with CHD [198]. The etiology of CHD is a multifactorial phenomenon. However, it is usually caused by the accumulation of fatty deposits (atheroma) on the walls of the arteries around the heart (coronary arteries) [199, 200]. On the other hand, details of the contribution of environmental factors to CHD remain unknown. However, studies have shown that maternal diabetes mellitus (matDM) is a common environmental risk factor for CHD [201].

Additionally, a Significantly different expression of more than 100 circulating miRNAs, including miR-1275, miR-142-5p, miR-34a, miR-4666a-3p, and miR-3664-3p, in the peripheral blood of pregnant women carrying fetuses with abnormal heart growth compared to women with normal pregnancies has also been reported [202]. Thus, these results confirm that circulatory exosomes or circulating miRNAs in pregnancy regulate fetal heart growth and provide new insights into CHD prevention, diagnosis, and treatment [8]. On the other hand, the transfer of exosomal miRNAs can play an important role in protecting against myocardial ischemia and inhibiting myocyte death by suppressing genes involved in cell death [203]. For example, Jin et al., as the first study, showed that exosomal expression of maternal serum miR-146a-5p was associated with fetal ventricular septal defects (VSD), the main type of CHD accounts for 20 to 30% of CHD cases [114].

At present, due to advances in medical care, some medications help protect the heart against further defects or damage, such as prostaglandins and prostaglandin inhibitors, and aspirin is used to keep the fetal artery open, catheter or surgical methods. Percutaneous valve implantation has recently shown promising results [198].

Recently, exosomes have emerged as a novel in the treatment of CHD. The delivery of intracellular signaling mediators of exosomes to transmit useful signals to damaged tissues is particularly important in cardiac regenerative medicine [204]. In particular, exosomes isolated from human embryonic stem cell-derived mesenchymal stem cells (ESC-MSCs) were used to treat ischemia/reperfusion (I/R) injury [205], improvements in cardiac function, and reduce infarct size [206].

A successful exosome therapy for heart repair probably involves a combination of exosomes that contain cargo to reduce cardiac fibrosis, induction of angiogenesis, and improvement of heart function. However, further studies in patients are necessary to optimize the dose and method of administration and study the immune response and side effects.

#### Neural tube defects

Neural tube defects (NTDs) are the second most common congenital abnormalities of the CNS*,* caused by a defect in neural tube closure during neurulation in early embryonic development, including anencephaly, spina bifida, and encephalocele, in the 3rd and 4th week of gestation [207]. One of the most common types of NTD is spina bifida aperta (SBA), which causes urinary and neurological complications [208]. The incidence of NTDs has declined significantly and now occurs in approximately 0.5–2 per 1000 pregnancies worldwide [209]. Although the pathogenesis of NTD is not yet fully understood, studies suggest that it has a multifactorial origin that includes genetics and environmental factors. The most common NTDs are spina bifida and anencephaly, but the clinical spectrum includes craniorachischisis, encephalocele, and iniencephaly [210]. Epigenetic changes, such as histone changes and DNA methylation, folate metabolites, and related enzymes, are involved pathogenesis of NTDs [8, 211]. Folic acid deficiency is a significant cause of NTDs, and reduced gene methylation plays an essential role in the mechanisms underlying the effect of folic acid on NTDs [212].

On the other hand, some potential targets of six miRNAs such as CBS, DNMT3A, FPGS, and MTHFD2 are involved in folate methylation or homeostasis. Thus, misrepresentation of miRNA may help NTDs by modulating folate methylation or metabolism [213]. Highly sensitive and specific biomarkers in maternal serological analysis for early detection of NTDs have not yet been identified [214]. However, different protein expression levels in serum exosomes of pregnant women during the 14–20 weeks of gestation can be used for earlier clinical screening and diagnosis of NTDs. For example, Wang et al. showed that the expression of proteins DNM2 and CORO1A was significantly reduced in pregnant women carrying NTD fetuses compared with a normal group [208]. In addition, in a recent study, Kumar et al. Showed that in spina bifida, PMSC-derived exosomes with neuronal survival-related proteins such as galactin-1 could play a protective role in nerve function [215]. Therefore, exosomes may be a potential way for early prenatal diagnosis of fetal neurological diseases.

On the other hand, maternal diabetes and obesity (BMI ≥ 30) are other known risk factors for NTDs. Human epidemiological data suggest that folic acid supplementation may reduce the risk of NTD in diabetic pregnancy [216], while the risk of increased BMI was independent of maternal folate status [217]. Several tests included prenatal "triple markers" which measured the maternal level of human chorionic gonadotropin (hCG), alpha-fetoprotein (AFP), estriol, and testing the level of AFP and acetylcholinesterase in amniotic fluid as well as chromosomal abnormalities, are used for screening and detecting neural tube defects. Imaging techniques such as prenatal anomaly scans also help identify a variety of NTDs [218].

## Exosomes and recurrent pregnancy loss

Recurrent pregnancy loss (RPL) is one of the most common reproductive disorders, defined as at least two or more pregnancy losses before 20–24 weeks of pregnancy, occurs in 1–2% of women [219, 220]. The etiology is considered to be multifactorial. Chromosomal abnormalities (Aneuploidy and polyploidy) are common problems in RPL [221]. Other probable or possible etiologies include uncontrolled diabetes mellitus, thyroid disorders, inherited uterine anatomic abnormalities, endometrial dysfunction, acquired and inherited thrombophilic disorders, abnormal HLA‐G expression, immunologic abnormalities, environmental factors, maternal age, and socioeconomic conditions [222–226]. In addition, some infections, including Listeria monocytogenes, Toxoplasma gondii, Herpes simplex virus (HSV), measles, and cytomegalovirus, are suspected causes of sporadic spontaneous pregnancy loss [227]. Among the immunological factors, antiphospholipid antibody syndrome (APS) is the most common treatable cause of recurrent miscarriage and is diagnosed in 15–20% of women with recurrent miscarriage [228].

On the other hand, Recent studies have shown that pregnancy exosomes (pEXO) play an important role in maternal–fetal immune tolerance, essential for a successful pregnancy [5]. For example, exosomes derived from endometrial epithelial cells promote fetal attachment during implantation by miR-30d-dependent upregulation of integrins or activating the focal adhesion kinase (FAK) signaling pathway [96]. Another study shows that diapause of exosomes derived from endometrial epithelial cells enriched with miR-let-7 can protect the fetus from collapse [5]. Interestingly, the study of Mincheva-Nilsson et al. showed that during a normal pregnancy, the level of exosomes increased compared to on-pregnant women, and placenta-derived exosomes which bear FasL (and possibly MIC) contribute to maintaining the state of immune privilege by suppressing the maternal immune response against a semi-allogenic fetus [229]. In contrast, women with premature labor have lower numbers of circulating placenta-derived exosomes, and FasL levels on exosomes reduced significantly [230, 231]. In previous studies, it has been reported that EV cargo increases in pregnant women compared to nonpregnant (~ 20 times more), with an increased gestational age that can be seen in the first trimester of pregnancy, starting as early as the sixth week [1], hence, EV levels and cargo can help modulate the mother's immune response to the fetus.

Moreover, MSC-derived exosomes have been found can deliver functional proteins and microRNAs that modulate the immune response [232]. Studies have shown that in treatment with exosomes in female mice, macrophages at the mother-fetus interface increased IL-10 release and decreased IL-12 secretion, indicating the anti-inflammatory type M2, but had no effect on the expression of CD86 and CD206. On the other hand, Exosome injection downregulated the production of TNF-α and IFN-γ and upregulated IL-4 and IL-10 [233]. These results showed that treatment of MSC-derived exosomes suppresses the Th1 immune response and facilitates the Th2 immune response, promoting Th2 dominance in the immune environment and beneficial for pregnancy. The conclusion from these findings is that treatment of MSC-derived exosomes modulates macrophage activity and T cell function at the interface of mother and fetus, which provides a therapeutic potential for RPL therapy.

## Exosomes in male and female infertility

The role of exosomes in male and female infertility has recently received more attention. At least one in six couples worldwide has infertility; 20–35% are due to female infertility, 20–30% are due to male infertility, and 25 to 40% are related to both sexes' infertility [234]. Seminal exosomes comprise 3% of total seminal plasma proteins. They are composed of several proteins that play a vital role in acrosome reactivity, sperm motility, and fertilization [235]. These vesicles contain mRNAs and miRNAs that can be transferred to another cell and functional in this new location. This RNA is called "exosomal shuttle RNA". Several studies found an association between the quality of sperm and aberrant miRNA levels, both in whole seminal plasma and exosomes [236, 237]. It has been reported that miRNA seminal exosomes modulate the male reproductive tract to support fetal growth [238]. These features support the idea that the study of exosomal miRNA content can be used as a specific indicator of alterations of the organ/cells that released them. The microRNAs studied in male infertility are miR-34, miR-122, miR-146b-5p, miR-181a, miR-374b, miR-509-5p and miR-513a-5p [236]. Similarly, dysregulation of miR-449 due to impaired motility of the spermatozoa was also linked to male infertility [239]. These microRNAs could potentially be used as diagnostic biomarkers for idiopathic male infertility and the development of treatment tools. In addition, aberrant expression of exosomal annexin II protein can affect sperm function and affect fertilization [235]. On the other hand, exosomes are secreted from various parts of the female reproductive tract, including the uterus, endometrium, oviducts epithelium, trophoblastic cells, and placenta. As mentioned above, due to the ability of exosomes in transporting signaling molecules, they can also play a role in female infertility [240]. Signaling pathways such as wingless signaling pathway (Wnt), transforming growth factor-beta (TGF-β), neurotrophin, insulin signaling pathways, mitogen-activated protein kinase (MAPK), epidermal growth factor receptor (ErbB) pathways, and pathways associated with ubiquitin inside exosomes [240]. Recently demonstrated that exosomes can be released from first-trimester placental mesenchymal stem cells (pMSC) under stressful conditions. For example, placental cells in response to changes in environmental conditions, such as low oxygen tension (1%) and high glucose concentrations (25 mm), and high concentration of TNF-α, secrete maximal exosomes, which modify the phenotype of recipient cell [241]. As a result, increased exosomes release in response to challenging environmental conditions may disrupt the Th1/Th2, Th17/Teg-2 cytokine balance. Notably; this balance is necessary for placentation, normal implantation, and successful pregnancy outcome. Placental exosomes express the proapoptotic molecules FasL and TRAIL that play an important role in infertility [242]. In the end, it can be concluded that many different functions attributed to these vesicular structures emphasize their importance, and this pluripotency can be considered as an important aspect of the normal process of reproduction.

## Conclusion

It can be concluded that exosomes play a significant role in pregnancy, egg implantation, and embryo formation. Exosomal content can play a role in pregnancy disorders and diseases of this period. Exosomes' potential diagnostic and therapeutic properties promise a bright future for using these compounds in all diseases, especially pregnancy disorders. Accurate knowledge of the biology and functional properties of these compounds can empower us in their therapeutic and diagnostic uses.

## Data Availability

The datasets used and/or analyzed during the current study are available from the corresponding author on reasonable request.

## Abbreviations

AD: Alzheimer's disease
AFP: Alpha-fetoprotein
APP: Amyloid precursor protein
ARPC2: Actin related protein complex 2
APS: Antiphospholipid antibody syndrome
BW: Birth weight
BMI: Body mass index
BMP: Bone morphogenetic protein
CHT: Chronic hypertension
CLIC4: Chloride intracellular channel protein 4
CVTs: Chorionic villous trophoblasts
cfDNA: Cell-free DNA
DS: Down syndrome
EVs: Extracellular vesicles
EVT: Extravillous trophoblast
ErbB: Epidermal growth factor receptor
ESCRT: Endosomal sorting complex required for transport
FGR: Fetal growth restriction
GDM: Gestational diabetes mellitus
HA: Hyaluronic acid
HMGCS1: 3-Hydroxy-3-methylglutaryl-CoA synthase 1
hCG: Human chorionic gonadotropin
HATs: Histone acetyl transferases
HSV: Herpes simplex virus
ILVs: Intraluminal vesicles
IRS1: Insulin receptor substrate
LMWH: Low molecular weight heparin
LPS: Lipopolysaccharide
MVs: Microvesicles
MVBs: Multivesicular bodies
NIPT: Nicht-invasive prenatal tests
NK: Natural killer cells
NGS: Next-generation sequencing
NTDs: Neural tube defects
PKM2: Pyruvate kinase type M2
UC-MSCs: Umbilical cord mesenchymal stem cells
ULF: Uterine luminal fluid
PdE: Placental-derived exosomes
RL29: Ribosomal protein L29
PPP6: Serine/threonine protein phosphatase 6
PPAR: Proliferator-activated receptor
PLAP: Placental-type alkaline phosphatase
PCP: Planar cell polarity
RPL: Recurrent pregnancy loss
pPROM: Preterm premature rupture of membranes
Shh: Sonic Hedgehog
TNIL: Term not in labor
TL: Term in labor
TSA: Trichostatin A
VEGF: Vascular endothelial growth factor
